# Neuromodulation of Attentional Control in Major Depression: A Pilot DeepTMS Study

**DOI:** 10.1155/2016/5760141

**Published:** 2015-12-28

**Authors:** Jodie Naim-Feil, John L. Bradshaw, Dianne M. Sheppard, Oded Rosenberg, Yechiel Levkovitz, Pinhas Dannon, Paul B. Fitzgerald, Moshe Isserles, Abraham Zangen

**Affiliations:** ^1^Monash Alfred Psychiatry Research Centre, Central Clinical School, The Alfred and Monash University, Prahran, VIC, Australia; ^2^School of Psychological Sciences and Monash Institute of Cognitive and Clinical Neurosciences, Monash University, Clayton, VIC 3800, Australia; ^3^Department of Neurobiology, The Weizmann Institute of Science, Rehovot 76100, Israel; ^4^Monash Injury Research Institute, Monash University, Clayton, VIC 3800, Australia; ^5^Beer Yaakov Mental Health Center Affiliated to Sackler School of Medicine, University of Tel Aviv, Israel; ^6^The Emotion-Cognition Research Center, Shalvata Mental Health Care Center, Hod HaSharon, Israel; ^7^Hadassah-Hebrew University Medical Center, Jerusalem, Israel; ^8^Department of Life Sciences, Ben Gurion University, Beer Sheva 84105, Israel

## Abstract

While Major Depressive Disorder (MDD) is primarily characterized by mood disturbances, impaired attentional control is increasingly identified as a critical feature of depression. Deep transcranial magnetic stimulation (deepTMS), a noninvasive neuromodulatory technique, can modulate neural activity and induce neuroplasticity changes in brain regions recruited by attentional processes. This study examined whether acute and long-term high-frequency repetitive deepTMS to the dorsolateral prefrontal cortex (DLPFC) can attenuate attentional deficits associated with MDD. Twenty-one MDD patients and 26 matched control subjects (CS) were administered the Beck Depression Inventory and the Sustained Attention to Response Task (SART) at baseline. MDD patients were readministered the SART and depressive assessments following a single session (*n* = 21) and after 4 weeks (*n* = 13) of high-frequency (20 Hz) repetitive deepTMS applied to the DLPFC. To control for the practice effect, CS (*n* = 26) were readministered the SART a further two times. The MDD group exhibited deficits in sustained attention and cognitive inhibition. Both acute and long-term high-frequency repetitive frontal deepTMS ameliorated sustained attention deficits in the MDD group. Improvement after acute deepTMS was related to attentional recovery after long-term deepTMS. Longer-term improvement in sustained attention was not related to antidepressant effects of deepTMS treatment.

## 1. Introduction

Major Depressive Disorder (MDD) is a debilitating and chronic psychiatric disorder. While depression is primarily characterized by mood disturbances, impaired attentional control is increasingly recognized as a cardinal feature of depression and is included in the diagnostic criteria of MDD as “an impaired ability to think or concentrate” [1]. Attentional deficits have been identified in patients with MDD [2–7] and are associated with increased depressive symptoms [8] and a heightened vulnerability to relapse [9]. Neuroimaging studies have demonstrated that these attentional processes in depressive populations are mediated by the fronto-parietal-limbic circuitry [6, 8, 10, 11], the same circuitry implicated in the pathophysiology of depressive disorders [12–16]. Therefore, there appears to be an intricate relationship between these attentional deficits and the pathophysiology of MDD; however, the neurobiological mechanisms underlying these attentional processes in MDD remain poorly understood.

Over the last decade, application of neuromodulatory brain stimulation techniques, such as transcranial magnetic stimulation (TMS), has emerged as a promising tool in treating clinical symptoms of treatment resistant depression (TRD) [17–20]. Repetitive transcranial magnetic stimulation (rTMS) involves application of a rapidly time variable magnetic field, delivered via an electromagnetic coil held above the patient's scalp, designed to either stimulate or disrupt neuronal activity in specific cortical regions [21–23]. These neuromodulatory effects on the frontal cortex may persist beyond the cessation of stimulation and lead to alterations in functionally connected regions [24–27] and may be associated with long-lasting alterations in neuroplasticity of the frontal regions [28–31].

Standard TMS techniques utilize a figure-8 coil which enables direct stimulation of superficial cortical areas [32, 33]. Standard TMS has been found to elicit moderate antidepressant effects in patients with MDD relative to sham [20, 34–36] when high-frequency (5–10 Hz) rTMS is applied to the left prefrontal cortex (PFC) to increase cortical excitability and low-frequency (1 Hz) rTMS to the right PFC [17]. These findings complement the identified cerebral asymmetry of the PFC in affective disorders presenting as reduced excitability in the left dorsolateral prefrontal cortex (DLPFC) and increased excitability in the right [37, 38]. While the standard rTMS techniques are capable of stimulating hypoactive frontal regions of depressive patients [15, 39–42], they are unable to directly stimulate the deeper cortical structures also implicated in the pathophysiology of depression, which are interconnected with both the dorsal and the ventral lateral prefrontal cortices [43–46]. This limitation led to the newly developed deep TMS (deepTMS) H-coil [33] which is able to safely modulate cortical excitability of deeper neural circuits [32, 33]. Similar to standard TMS, these deepTMS techniques target hypoactivity within the DLPFC [32, 33, 47] while stimulating deeper cortical structures within the fronto-parietal-limbic circuitry in treating depressive disorders [45, 46, 48, 49]. Therefore, delivery of repetitive TMS (rTMS) to the DLPFC targets the same compromised circuitry which correlates with impaired attentional control in MDD [6, 8, 10]. Accordingly, it is possible that deepTMS, in modulating cortical excitability of the deeper cortical and subcortical regions, may also induce concomitant improvements in attentional symptoms of depression. However, while TMS techniques (both standard and deepTMS) have been widely examined for its therapeutic value and ability to ameliorate mood symptoms in depressive populations, far fewer studies have examined the effects of TMS on cognitive features of depression [50–52].

These preliminary studies have examined potential TMS-induced attentional enhancements in healthy controls [53–56] or depressive populations [57–59]. Results from these studies suggest that acute administration of high-frequency of standard rTMS to the left DLPFC leads to improved attentional function [53, 54] and related increases in activity in the frontal regions of healthy controls [53]. Notably though, these preliminary studies were conducted in small samples and not all studies are in agreement over the attentional enhancing effects of rTMS to the left DLPFC in healthy controls [56]. Regardless, they indicate that high-frequency rTMS to the DLPFC is indeed capable of stimulating the frontal cortex and influencing the brain circuitry subserving attentional control.

In depressive patients, low-frequency standard rTMS to the right DLPFC enhanced attentional task performance in acutely depressed patients, while low-frequency rTMS to the left DLPFC resulted in impaired attentional performance in patients with remitted depression [59]. Following this, a series of studies explored the acute (1 session) and long-term (10 sessions) effects of high-frequency standard rTMS delivered to the left DLPFC. A single session of high-frequency standard rTMS delivered to the left DLPFC was related to significant improvements in the patients' performance in attentional control (task-switching paradigm); however, these improvements in attentional control occurred independently of changes in mood symptoms of depression [57]. The same research group also examined the effect of high-frequency standard rTMS to the left DLPFC on performance on the negative affective priming task. In this study, though a single session of rTMS did not improve inhibitory attentional processing, a relationship was observed between treatment response following 10 sessions of rTMS and improvement in inhibitory control [60]. In a preliminary deepTMS study, spatial attention was examined using the Rapid Visual Processing task and it was found that while treatment-over-time performance effects were not significant, the MDD patients performed similarly on the visual spatial attention task to controls following long-term rTMS treatment [61]. While these studies do not demonstrate a direct effect of long-term rTMS on attentional control, they provide initial evidence of the relationship between high-frequency rTMS-evoked improvements in attentional control and symptoms of depression, with these attentional modifications being a possible predictor of treatment response.

Therefore, the current study aimed to expand on these preliminary standard rTMS and deepTMS studies by examining both the acute and the long-term effects of applying high-frequency repetitive deepTMS to the DLPFC on the Sustained Attention to Response Task (SART) [62] in a depressive population. This was followed by an examination of the potential relationship between the acute and longer-term attentional improvements and that between deepTMS-evoked long-term attentional improvements and clinical response to deepTMS treatment. We predicted that both acute and long-term high-frequency repetitive deepTMS to the DLPFC would attenuate attentional deficits associated with MDD. Additionally, it was anticipated that the neuromodulatory attentional effects of acute deepTMS would relate to the longer-term neuroplasticity-related attentional effects of long-term deepTMS applied to the frontal brain regions. Finally, we proposed that these deepTMS-evoked improvements in attentional control may occur independently of improvements in clinical response, which would suggest that these attentional improvements are a product of deepTMS and do not necessarily occur as a side-effect of clinical improvement.

## 2. Methods

The current study was part of a larger clinical study (clinicaltrials.gov, NCT00460902 and NCT00577070) approved by institutional and national review board (IRB) committees. The study was conducted in collaboration with the Department of Psychiatry, Hadassah Medical Center, Hebrew University, Jerusalem, Beer-Ya'acov Mental Health Centre, Beer-Ya'acov, and Shalvata Mental Health Center, Hod HaSharon, Israel. Patients signed a detailed informed consent form prior to study enrolment; they were informed that participation was voluntary and they could withdraw at any time without prejudice. Active enrolment ran from July 2008 to April 2009.

### 2.1. Participants

#### 2.1.1. Major Depression Sample

Twenty-one MDD patients were assessed at baseline and completed the short-term component of the study. To determine suitability, the screening procedure included a psychiatric and medical interview conducted by a psychiatric clinician. Main criteria for inclusion were as follows: clinical diagnosis of nonpsychotic Major Depression Disorder in patients who did not respond to at least two antidepressant medications, provided in appropriate doses and duration, in the current episode, and no coexisting DSM-IV axis I or major axis II disorder. Patients completed the Hamilton Depression Rating Scale (HDRS) [63], a 24-item clinical interview, and those with a HDRS score >21 and aged 18–65 years were recruited for the study. Throughout the study, patients were administered the Beck Depression Inventory (BDI) [64], a 21-item self-report measure to quantify depression severity. A subgroup of 13 MDD patients continued treatment and participated in the long-term component of the study. Throughout the study, no change was made to antidepressant treatment with only limited use of hypnotic or anxiolytic medication (up to 2 mg/day lorazepam or equivalent) for treatment-emergent insomnia or anxiety. Current antidepressant treatment of the MDD patient group includes selective serotonin reuptake inhibitors (SSRI: 33% of patients), tricyclic antidepressant (TCA: 33% of patients), serotonin and norepinephrine reuptake inhibitors (SNRI: 28% of patients), and serotonin antagonist and reuptake inhibitors (SARI: 6% of patients).

#### 2.1.2. Healthy Control Sample

Twenty-six healthy control subjects (CS), without any current or previous major medical/psychiatric illness, were recruited through local advertisements. They were paid 100 NIS (approximately $30 US) to participate in the study (covering travel costs). At screening, CS completed a general demographic questionnaire, as well as the BDI to screen for potential confounding levels of depressive symptoms (BDI scores ≥9 were excluded from the study). Relevant demographic and participant characteristics for MDD and CS are summarized in Table 1.

### 2.2. Procedure Overview

**Figure 1:**
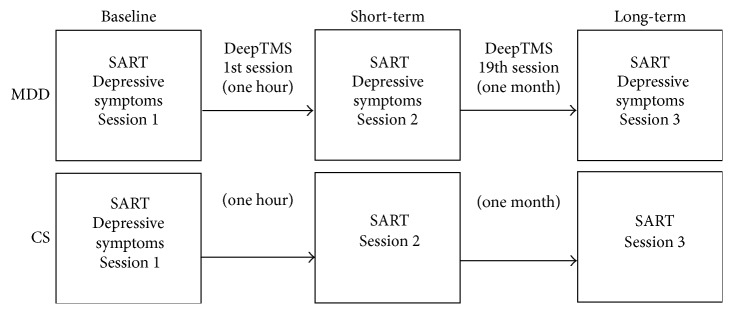
Timeline of the study procedure. In the Major Depressive Disorder (MDD) group, cognitive performance on the Sustained Attention to Response Task (SART) was assessed at three time-points. Session 1 (baseline), Session 2 after a single application of deepTMS (short-term), and Session 3 immediately prior to the 20th application of deepTMS (long-term). To control for the presence of a practice effect, cognitive performance was also evaluated at the same three time-points in the control subjects (CS); however, CS were not administered the deepTMS treatment.

The deepTMS procedure (4 weeks) consisted of daily deepTMS sessions scheduled in a 5-day sequence each week. A total of 20 sessions were conducted. Baseline evaluations were conducted prior to the first deepTMS treatment, short-term cognitive evaluation was assessed directly following the first deepTMS treatment session, and the long-term evaluation was examined prior to the 20th treatment session. MDD patients completed basic demographic and depression severity (BDI) questionnaires at baseline (prior to the initial TMS treatment). Depression severity (BDI) was again assessed immediately prior to the 20th TMS session. In the MDD group, cognitive performance was evaluated at three time-points: baseline, after the first deepTMS session (short-term), and immediately prior to the 20th TMS session (long-term). Throughout the course of the study, patients were under direct monitoring, and any adverse effects or complaints were immediately recorded and responded to by qualified on-site psychiatrists. The control group also completed the basic demographic and depressive symptom (BDI) questionnaires at baseline. Cognitive performance was evaluated at the same three time-points: baseline, one hour after baseline, and one month after baseline (Figure 1). However, the control group was not administered the deepTMS treatment. Rather, data from the controls were used as a comparative tool to compare the baseline level of cognitive performance in MDD patients relative to CS and, further, to assess any practice effect due to repeated administration of the cognitive tasks.

#### 2.2.1. Baseline

Following baseline screening, cognitive performance for both MDD patients and CS was evaluated by administering the Sustained Attention to Response Task (SART) [62], a computerized cognitive task which uses E-prime V1 technology (Psychology Software Tools). Participants were seated in a quiet, well-lit room, 30 cm from the 17-inch computer screen. Task instructions were presented in Hebrew. Each task began with a short demonstration of the task requirements. All participants were native speakers of Hebrew.

#### 2.2.2. Short-Term Cognitive Evaluation

Following screening and the baseline cognitive evaluation, MDD patients were administered a single treatment of deepTMS. Prior to stimulation, earplugs were inserted to prevent any potential adverse effects on hearing. To determine the appropriate stimulation parameters, single pulse stimulation was applied to the motor cortex, and Motor Threshold (MT), the point at which a minimum electric field would induce a noticeable motor response (i.e., twitching of the contralateral finger muscles) in 3 out of 5 trials, was measured. Next, the coil was moved 6 cm anterior of the motor spot and placed over the PFC ready for the deepTMS treatment session. Consistent with previous deepTMS studies in clinically depressed patients [61, 65–67], the high-frequency (20 Hz) deepTMS session included administration of 42 trains of pulses, with each train consisting of 40 pulses within 2 seconds at 120% of measured MT intensity, with an intertrain interval of 20 seconds (i.e., 1680 magnetic pulses over 15.5 minutes). Immediately following the initial deepTMS session, cognitive performance was reevaluated through readministration of the SART.

#### 2.2.3. Long-Term Cognitive Evaluation

The long-term trial consisted of five daily stimulation sessions (according to the protocol described in the short-term deepTMS section) per week, over 4 consecutive weeks. MT was measured daily, and the stimulation parameters were administered according to 120% of the daily measured MT (mean = 50.16; SD = 6.28). Prior to the 20th deep TMS session, cognitive performance was reevaluated for the third time through readministration of the SART.

### 2.3. Materials

#### 2.3.1. Deep Transcranial Magnetic Stimulation (DTMS)

The deepTMS stimuli were delivered using the Magstim Super Rapid Stimulator (Magstim, UK). The Magstim stimulator was connected to an extracorporeal device, the novel H-coil, which was positioned on the patients' scalp prior to the stimulation session. The H-coil consists of seven Shelamid 200 copper wires, insulated by two polyester layers, set tangentially to the surface of the scalp [33]. The inner frame of the H-coil is flexible to fit the variability in contour of the human scalp. The H1-coil is designed to stimulate deep prefrontal regions, preferentially the left hemisphere [32, 33, 47].

#### 2.3.2. Computerized Cognitive Tasks

*Sustained Attention to Response Task (SART).* For the SART, participants were asked to respond quickly and accurately to the presentation of single digits (1 to 9) with a button press, with the exception of the number “3” the target stimulus [62]. The stimuli appeared in black in the centre of white background, presented in a random order in a block of 297 trials, with 33 (i.e., 1 in 9 trials on average) possible no-go (number 3) responses. Each stimulus was presented for 150 ms, with varying interstimulus interval (ISI) durations (1000 ms, 1500 ms, and 1250 ms) randomly distributed throughout the session [68, 69]. The variable ISI was used to minimize speed accuracy trade-offs. Prior to recording, participants were administered 18-trial demonstration sequence, with 2 possible no-go trials presented randomly. Participants were informed that speed of response and accuracy were of equal importance. Reaction time (RT), commission errors (responding when you should withhold), omission errors (withholding when you should respond), and performance variability (individual variations in response time) were recorded.

### 2.4. Data Analysis

Comparability of MDD patients and controls was assessed using *χ* ^2^-tests for categorical and *t*-tests for continuous variables (Table 1). Stem-plots located extreme outliers (>±2.5 standard deviations (SD)), and outliers were brought to 2.5 SD of the mean. For all data which met assumptions of normality, tests were run at an alpha level of 0.05 (two tailed). In very few cases, there were violations of unequal variance (Levene's Test > 0.05); for those cases, statistics were run at a more conservative alpha level of 0.025 [70]. Homogeneity and sphericity assumptions were met.

In the cognitive tasks, mixed model ANOVAs were used to analyze both between-group differences (between MDD and CS) and changes in performance over time (between baseline and short-term/long-term). Cognitive performance on the SART (SART RT, performance variability and omission errors) was examined. To control for the potential covariance of SART RT on measures of commission errors [71, 72], two one-way between-groups ANCOVAs were used to explore group differences for commission errors. The first ANCOVA examined session differences in commission errors (baseline versus short-term/long-term) after adjusting for commission errors at baseline (controlling for SART RT as a covariate). The second ANCOVA assessed whether number of commission errors differed over time (sessions) between the two groups (again controlling for SART RT as a covariate). Change in number of commission errors was calculated by subtracting the baseline errors score from the session (short-term or long-term) commission error score. Repeated measures ANOVA was used to examine whether there was a change in depressive symptoms (BDI score) over time (between baseline and long-term deepTMS) in the MDD group. In MDD patients who completed all sessions of the study (*n* = 13), repeated measures ANOVA was used to examine whether there was a change in depressive symptoms (BDI score) over time (between baseline and long-term deepTMS).

Pearson's correlation examined potential associations between basic demographics, SART performance, and BDI scores, within each group separately. Additionally, we investigated whether significant changes in cognitive performance correlated with change in depression (BDI) levels. All data analyses were performed using SPSS version 15.

## 3. Results

### 3.1. Baseline Demographics

Group comparisons between the MDD group (short- and long-term cohorts) and CS identified significant differences at baseline in BDI across the two tasks but no group differences in age, gender, or education (Table 1). In the MDD group, there was considerable improvement in BDI scores following long-term deepTMS treatment relative to the baseline measures *F*(1,12) = 18.565, *p* = 0.001.

### 3.2. Baseline Cognitive Data across the SART

The MDD group made significantly more errors of omission and commission than CS, [*F*(1,45) = 9.712 and *p* = 0.003; *F*(1,44) = 7.41 and *p* = 0.009, resp.]. Performance variability was significantly greater in the MDD group relative to CS, *F*(1,45) = 7.985, and *p* = 0.007 (Table 2).

### 3.3. Effect of a Single Session of DeepTMS on SART

**Figure 2:**
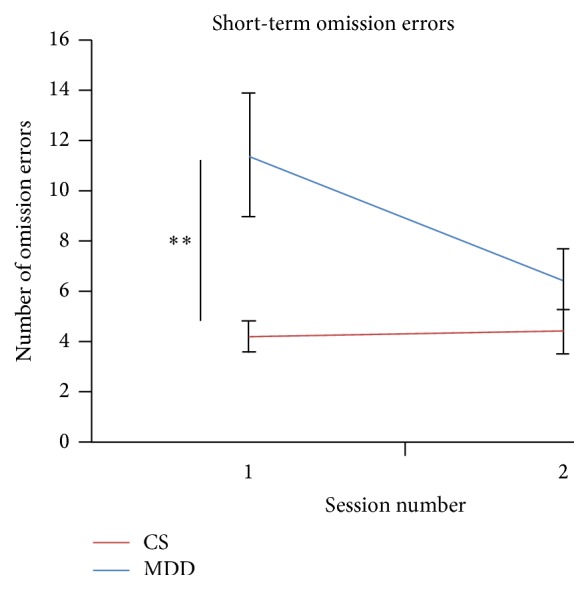
Adjusted group means and standard error of short-term omission errors from the Sustained Attention to Response Task for clinically diagnosed Major Depressive Disorder (MDD) patients and control subjects (CS). The significance differences reported relate to the post hoc analysis of interaction effects, ^*∗∗*^ *p* < 0.01.

There was a significant effect of group on omission error rate (*F*(1,45) = 8.5; *p* = 0.006) as well as a significant session effect (*F*(1,45) = 4.70; *p* = 0.036). There was also a significant interaction effect (*F*(1,45) = 5.41; *p* = 0.025) (see Figure 2), such that the MDD group committed more omission errors than the control group at baseline (*F*(1,45) = 9.712; *p* = 0.003) but this group difference was not significant at Session 2 (*F*(1,45) = 2.010; *p* = 0.163) (Table 3).

### 3.4. Effect of Long-Term Treatment of DeepTMS on SART

**Figure 3:**
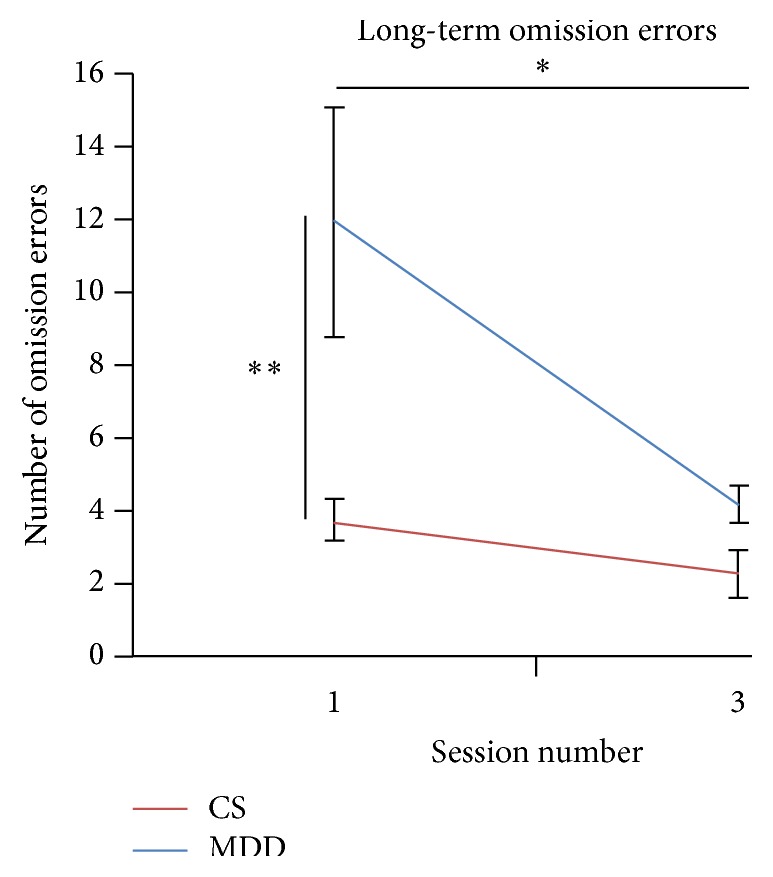
Adjusted group means and standard errors of omission errors from the Sustained Attention to Response Task across Sessions 1 and 3 for clinically diagnosed Major Depressive Disorder (MDD) patients and control subjects (CS). The significance differences reported relate to the post hoc analysis of interaction effects, ^*∗*^ *p* < 0.05, ^*∗∗*^ *p* < 0.01.

The MDD group committed more omission errors than the control group at baseline (*F*(1,34) = 11.149; *p* = 0.002) but this difference was not significant by session 3 (*F*(1,34) = 5.998; *p* = 0.02) (Figure 3). Although no significant interaction effect was observed, there was a significant improvement observed in the MDD group (*F*(1,34) = 5.192; *p* = 0.023) while no such improvement was identified in the CS group (*F*(1,44) = 2.609; *p* = 0.114) (Table 3). Additionally, the MDD patients exhibited a significant improvement in omission errors from Session 2 (short-term) to session 3 (long-term) (*F*(1,12) = 5.732; *p* = 0.034).

### 3.5. Correlational Data: Predictors of Long-Term Cognitive and Clinical Response in MDD Group

Improvement in omission errors observed after a single session of rTMS was positively related to long-term improvement of omission errors (*r* = 0.922, *n* = 13, and *p* = 0.0005).

Despite being near significance, the relationship between long-term improvement in omission errors and improvement in BDI scores in the MDD group was not significant (*r* = 0.442, *n* = 13, and *p* = 0.113).

## 4. Discussion

The present study is the first investigation to focus primarily on the potential for frontal deepTMS to reduce deficits of attentional control in patients with major depression. The study presented confirmatory evidence of executive deficits across the domains of attentional control and cognitive inhibition within the MDD population relative to CS. Following this, application of both acute and long-term high-frequency repetitive frontal deepTMS to the DLPFC was found to ameliorate sustained attention deficits in the depressive sample. Improvement in sustained attention after acute deepTMS for those with MDD was also strongly associated with attentional recovery after long-term deepTMS. Interestingly, these improvements in sustained attention were not directly related to clinical improvement following long-term deepTMS treatment.

### 4.1. Attentional Control and Cognitive Deficits in MDD

Confirming previous studies, the MDD group presented with impairments in attentional control (i.e., increased commission errors, omission errors, and performance variability) [73] and response inhibition (i.e., increased commission errors) [74] on the SART relative to CS.

### 4.2. Effect of rTMS on Attentional Deficits in MDD

The present study provided direct evidence for the first time of the beneficial effect of both acute and long-term administration of high-frequency repetitive deepTMS to the DLPFC in improving sustained attention (omission errors) within a depressive sample. These improvements are unlikely to be due to a practice effect as these same improvements were not demonstrated by the CS group upon repeated task administration. Additionally, by the end of the deepTMS treatment, any significant performance difference between the MDD and CS groups in sustained attention had dissipated.

#### 4.2.1. Short-Term Effect of rTMS on Attentional Deficits in MDD

As predicted, a single session of high-frequency repetitive deepTMS to the DLPFC was related to improvements in sustained attention in the depressive population. These findings are likely to reflect the ability of rTMS to upregulate cortical excitability in the frontal cortex [25, 27, 53] and functionally connected regions [26, 28]. This suggests that an acute session of deepTMS might be a useful tool in modulating the altered cortical networks associated with attentional deficits in depressive disorders. These results also complement those of standard TMS studies which have found that delivery of a single session of high-frequency rTMS to the left DLPFC to healthy controls leads to improved attentional control [53, 54]. Additionally, rTMS-evoked attentional improvements have been reported to be related to increased activity within the right DLPFC, dorsal anterior cingulate, right superior parietal gyrus, and left orbitofrontal cortex [53]. Similar studies with depressive populations have shown that low-frequency rTMS to the right DLPFC enhances attentional task performance (affective go/no-go task) in acutely depressed patients [59], and high-frequency rTMS delivered to the left DLPFC induces significant improvements in patients' performance in attentional control (task-switching paradigm) [57]. However, despite the promising results from these preliminary studies, there is currently no consensus regarding the exact effects of a single session of high-frequency rTMS applied to the DLPFC. A previous study observed no significant effects of delivery of rTMS on attentional control [60], while another study suggested that rTMS-evoked changes in attentional control only occurred in patients who responded to rTMS treatment [58]. Finally, and in contrast to all of the above findings, a third study observed a deterioration in divided attention following high-frequency rTMS to the DLPFC [56]. However, as all of these preliminary studies applied rTMS via the standard TMS coil, it is possible that the inability to directly stimulate the deeper cortical structures implicated in the neurobiology of attentional control [75] may partly account for the discrepancy in these findings. Consistent with this notion, the current study provides the first report of attentional enhancing effects of a single session of high-frequency repetitive deepTMS applied to the DLPFC in depressive patients.

#### 4.2.2. Long-Term Effect of rTMS on Attentional Deficits in MDD

Moreover, the current study also demonstrated evidence for longer-term effects of applying high-frequency repetitive deepTMS to the DLPFC in improving sustained attention in a depressive population. These improvements in sustained attention were reported* prior* to the application of the 20th treatment which allowed us to discern between the long-term and acute effects of deepTMS; therefore, these findings support the proposition that these attentional improvements may be associated with longer-lasting alterations in neuroplasticity of the frontal and interconnected regions [27, 29, 31, 76]. Our findings complement the previous assessment of the long-term visual spatial effects of high frequency repetitive deepTMS administered to the DLPFC of depressive patients [61]. In this study, visual spatial attention was measured by the Rapid Visual Processing task, and although the treatment-over-time effects were not significant, the MDD patients achieved similar levels of spatial attention as controls following long-term rTMS treatment. Previously, only a very small number of preliminary studies have examined longer-term attentional effects of delivering high-frequency standard rTMS to the left DLPFC on depressive patients, finding no evidence of attentional improvements [58, 60, 77]. As with the discrepancies in the short-term effects of rTMS reported above, it is possible that long-term deepTMS has a greater potential to induce attentional improvements in MDD population due to its capacity to induce changes in neuroplasticity in the deeper cortical and subcortical regions. Despite encouraging results reported by the current study, this assertion remains speculative as it is based on results from a relatively small sample size and, as such, future studies are required to provide further validation of this claim. Despite these limitations, the current study is the first to provide direct evidence of the beneficial effect of both short-term and long-term administration of high frequency frontal deepTMS in improving sustained attention within a depressive sample.

#### 4.2.3. Relationship between Short-Term and Long-Term rTMS-Induced Attentional Improvements in MDD

The current study also found that improvement in sustained attention following a single session of deepTMS was strongly associated with longer-term attentional recovery. Additionally, it was shown that these improvements were significantly greater following long-term deepTMS when compared with short-term deepTMS. We speculate that a single session of high frequency rTMS may induce a short-lasting increase in the release of dopamine within the frontal network [27, 76] which results in these more immediate attentional improvements. While, for long-term treatment, repeated short-lasting increases in excitability [27, 76] within the deep cortical regions may be associated with longer-lasting alterations in neuroplasticity of the frontal regions and functionally connected regions [28, 29, 31], thereby resulting in longer-term improvement of these attentional deficits. This is consistent with our findings that, firstly, the attentional performance following acute administration of deepTMS was strongly related to longer-term attentional improvements and, secondly, that the longer-term improvements were significantly better than the short-term effects.

### 4.3. Relationship between Improved Sustained Attention and Depressive Symptoms

The current study demonstrated significant improvement of depressive symptoms following long-term deepTMS. Interestingly though, there was no conclusive evidence of a significant relationship observed between rTMS-related improvements in sustained attention and the attenuation of clinical symptoms of depression. Therefore, while the results of the current study revealed that acute and long-term deepTMS are capable of attenuating deficits in sustained attention in MDD patients, it is possible that these attentional improvements may occur independently of the antidepressant effects of deepTMS. This finding is supported by Vanderhasselt et al. [57, 58] who reported that application of acute rTMS led to significant improvements in attentional control that were not related to improved depressive symptoms [57]. This lack of association could suggest that attentional enhancements may be directly induced by high-frequency repetitive deepTMS of the DLPFC and do not necessarily occur as a side-effect of improved clinical symptoms. However, our findings are preliminary and future studies are required to further elucidate the relationship between these cognitive enhancements and clinical improvements and whether both attentional and depressive symptoms of MDD could be simultaneously targeted using deepTMS.

### 4.4. Limitations

Despite these promising findings, we must be cautious in interpreting the results of the current study due to the relatively small sample size. In addition, although no significant correlations between cognitive performance, depressive symptoms, and type/dose of medication were identified, it is possible that antidepressant medication may have influenced the results. Moreover, even though cognitive functioning was assessed across the three time-points in the CS group to control for a potential practice effect, the present study was unable to evaluate the possible placebo effect of long-term treatment in the MDD sample. This represents a consistent dilemma in the development of psychiatric treatments, as long-term sham treatment cannot be used with the MDD populations due to its potential detrimental effect on the patients' health. To address this limitation, future studies should consider adding a MDD control group, which does not receive any TMS during participation over the three time-points as a more valid measure of the MDD practice effect. Another important consideration is the ethical impossibility of applying the deepTMS treatment to healthy controls and inducing possible neuroplasticity changes within the healthy brain. For this reason, the healthy controls were not administered deepTMS but, rather, were included to provide a baseline measure of group differences and to present an estimation of potential practice effects. Finally, while the current study examined the longer-lasting effects of deepTMS on cognitive performance, future studies should conduct follow-up studies to examine the long-lasting effects (i.e., 6 months after treatment).

### 4.5. Conclusions

In conclusion, the current study provided the first report of the acute and longer-term efficacy of high-frequency repetitive deepTMS applied to the DLPFC in attenuating attentional symptoms of depression. Acute improvements in sustained attention may relate to deepTMS-induced neuromodulation of cortical excitability of cortical and subcortical networks associated with attentional deficits in depressive disorders, while long-term deepTMS is more likely to induce longer-lasting alterations in neuroplasticity of the frontal brain regions underlying the attentional deficits. These improvements in attentional deficits were not significantly related to the antidepressant effects of deepTMS; therefore, it appears that these attentional improvements may be a product of deepTMS treatment and not merely a byproduct of improved clinical symptoms. It is anticipated that the ability of deepTMS techniques to attenuate these attentional deficits will lead to the implementation of improved treatment strategies to target the persisting attentional symptoms of major depression.

## Figures and Tables

**Table 1:** Description of demographic and clinical data of participants.

SART	Major Depressive Disorder participants	Healthy control participants	*p* value
	(*n* = 21)	(*n* = 26)	
Age (years)	44 (9)	39 (12)	0.095
Gender (M : F)	10 : 11	15 : 11	0.49
Education (years)	15 (3)	16 (3)	0.51
BDI Session 1	32 (9)	2 (2)	<0.0005

	(*n* = 13)		
BDI Session 3	21.08 (10)	NA	NA

SART: Sustained Attention to Response Task; BDI: Beck's Depression Inventory; NA: not applicable.

**Table 2:** Mean and standard deviation of short-term cognitive data of participants.

SART	Major Depressive Disorder participants (*n* = 21)	Healthy control participants (*n* = 26)
Reaction time S1 (ms)	427 (65)	413 (57)
Reaction time S2 (ms)	412 (64)	397 (52)
Performance variability S1	0.26 (0.06)	0.23 (0.032)
Performance variability S2	0.23 (0.05)	0.2 (0.039)
Omission errors S1	10 (9.86)	3.69 (2.8)
Omission errors S2	5.67 (4.80)	3.85 (4.00)
Commission errors S1	9.24 (7.17)	6.23 (3.07)
Commission errors S2	9.86 (8.67)	5.5 (3.34)

SART: Sustained Attention to Response Task; ms: milliseconds; S1: Session 1; S2: Session 2.

**Table 3:** Mean and standard deviation of long-term cognitive data of participants.

SART	Major Depressive Disorder participants	Healthy control participants
	(*n* = 13)	(*n* = 23)
Reaction time S1 (ms)	428 (76)	411 (60)
Reaction time S3 (ms)	412 (75)	390 (51)
Performance variability S1	0.27 (0.06)	0.23 (0.03)
Performance variability S3	0.23 (0.043)	0.19 (0.04)
Omission errors S1	11.85 (11.24)	3.7 (2.75)
Omission errors S3	4.15 (1.95)	2.28 (3.19)
Commission errors S1	10.23 (8.35)	6.22 (3.26)
Commission errors S3	9.77 (8.22)	5.57 (4.00)

SART: Sustained Attention to Response Task; ms: milliseconds; S1: Session 1; S3: Session 3.
